# Allied health in the matrix: governance structures and workforce experience across two hospital eras

**DOI:** 10.1108/JHOM-04-2025-0212

**Published:** 2026-03-11

**Authors:** Gemma Turato, Rosalie Boyce

**Affiliations:** The School of Health, University of the Sunshine Coast, Sunshine Coast, Australia; Department of Allied Health, Sunshine Coast Hospital and Health Service, Birtinya, Australia; Department of Allied Health, South West Healthcare, Warrnambool, Australia; University of Queensland, Brisbane, Australia

**Keywords:** Allied health, Public hospital, Clinical matrix structure, Governance

## Abstract

**Purpose:**

This study contributes to international debates in health services management by examining how clinical matrix governance structures shape the workplace experiences of allied health professionals (AHPs) in public hospital bed-based services. Through a comparative analysis of two Australian hospital case studies conducted 26 years apart, it explores how and why governance tensions persist or evolve across institutional eras.

**Design/methodology/approach:**

Two qualitative case studies with comparable methodologies and contexts were analyzed. Each involved semi-structured interviews with managers and senior clinical leaders across allied health, nursing, medicine and administrative domains. A cross-case comparative analysis assessed how changes in organizational structure shaped AHPs' experiences and perceptions of their work environments.

**Findings:**

Sustaining a clinical matrix structure in acute and subacute hospital bed-based settings imposed complex governance demands that adversely impacted human resource management, operational requirements, budgetary control and professional morale among AHPs. These tensions were consistently associated with reduced job satisfaction and perceived inefficiencies.

**Research limitations/implications:**

The research is 26 years apart, but not a true longitudinal study, but more of a comparison decades apart. One limitation is that the study was conducted with managers and leaders only and did not gain the perspectives of front-line clinical staff.

**Practical implications:**

By comparing governance in specific environments across decades, this research provides hospital executives and policymakers with evidence-based insights into the risks and opportunities of clinical matrix structures for AHPs and offers actionable recommendations to enhance workforce sustainability, operational efficiency and patient care outcomes.

**Social implications:**

The allied health workforce often feel less valued than medical and nursing, particularly during re-structures in which they are placed into design's that often do not meet their needs from a governance perspective, including their social identity needs such as professional identity. This is often disrupted and overlooked by administrators during organizational re-design processes.

**Originality/value:**

Despite widespread adoption of matrix models, their long-term implications for allied health governance and workforce well-being remain underexplored. This study offers rare insight into how structural design affects AHPs' morale, coordination and professional voice, highlighting the need for governance models that better align with allied health's operational and professional requirements.

## 1. Introduction

Allied health professionals (AHPs) are essential to inpatient care yet are frequently marginalized in hospital governance arrangements, especially when organizations adopt clinical matrix models that create dual reporting and ambiguous control (Galbraith, 1971; Greenwood and Miller, 2009). AHPs, distinct from medicine, dental and nursing, play a critical role in interprofessional teams, focusing on minimizing risk, reducing hospital stays and ensuring effective discharge and follow-up care (Allied Health Professions Australia, 2024). Their roles include therapy (e.g. physiotherapy, psychology), diagnostics (e.g. radiography) and scientific services (e.g. pharmacy) (Turnbull *et al*., 2009). Effective governance mechanisms tailored to AHPs needs are crucial for enabling optimal performance, interprofessional collaboration and quality patient care (Braithwaite *et al*., 2005; Hastings *et al*., 2014; Law and Boyce, 2003; Mickan *et al*., 2018; Turato *et al*., 2023). This paper examines how clinical matrix governance structures shape AHPs workplace experiences in bed-based public hospitals and whether governance tensions persist across different organizational eras.

Healthcare systems globally face significant challenges, including rising costs due to chronic diseases, an aging population, inequitable access, workforce shortages and constrained budgets (Amalberti *et al*., 2016; World Health Organization, 2024). These pressures drive organizational redesigns intended to improve coordination and efficiency (Kajamaa and Hurmelinna-Laukkanen, 2022). Such redesigns include Integrated Care Systems in the United Kingdom (Care Quality Commission, 2025; NHS England, 2022), regional health authorities in Canada (Canadian Medical Association, 2025) and matrix arrangements in large academic medical centers in the United States (Wartman, 2024), each offering distinct governance trade-offs for allied health (i.e. integration vs profession-specific oversight; local responsiveness vs centralized control). These international variations illuminate recurring tensions between managerial and professional imperatives that shape how AHPs experience accountability, resource allocation and professional identity (Braithwaite *et al*., 2005). By comparing governance environments across decades, this research offers hospital executives and administrators evidence-based insights into the risks and opportunities of clinical matrix structures for AHPs and provides actionable recommendations for designing governance models that enhance workforce sustainability, operational efficiency and patient care outcomes (Turato *et al*., 2024).

Past literature has demonstrated that structural changes can negatively affect AHPs if poorly planned and executed (Boyce, 2001; Law and Boyce, 2003; Robinson and Compton, 1996; Turato *et al.*, 2022, 2023, 2024). Additionally, poor governance and organizational restructuring contribute to job dissatisfaction, early retirements and recruitment challenges among AHPs. Studies highlight that workplace structures and career development opportunities significantly impact retention of AHPs (Callan *et al*., 2007; Couch *et al*., 2021; Hewko *et al*., 2019). A survey of 1,005 US healthcare facilities in 2022 revealed that 85% faced allied health workforce shortages, reflecting a global issue (Roth *et al*., 2024). The World Health Organization predicts a shortage of 12.9 million healthcare workers by 2035, underscoring the urgency of addressing governance and workforce challenges (Truth, 2013). Hence, decision-makers and researchers must collaborate to understand how governance structures impact AHPs and develop evidence-based approaches to minimize undesirable outcomes (Mickan *et al*., 2018; Turato *et al*., 2023, 2024).

To address the above issues in their jurisdiction, the New South Wales (NSW) government in Australia developed a governance framework with the aim to address longstanding gaps in allied health governance. This framework emphasizes strategic, operational, clinical and professional governance domains to enhance AHPs capacity for service efficiency and innovation. Specifically, these domains support clearer leadership accountability, streamlined functional structures, robust safety and quality systems, and profession-specific standards. Together, these mechanisms aim to reduce duplication of effort, improve interprofessional coordination and enable more responsive models of care such as integrated service delivery, advanced scope roles and data-informed decision-making. However, governance variability and inconsistent clinical oversight remain persistent challenges that limit the full realization of these benefits (NSW Ministry of Health, 2023).

The use of historical comparison is a deliberate methodological choice that strengthens the originality of this study. Scally and Womack (2004) emphasize that public health research benefits from looking backwards, as historical analysis reveals continuities that shape present practice as much as innovations that mark change. Applying this lens to allied health governance enables us to separate structural tensions that persist across decades from those that are context-specific. In doing so, the study highlights how professional identity and managerial logics endure despite shifting organizational environments. This historical perspective therefore provides a deeper understanding of why certain governance challenges remain resistant to reform.

This study compares two Australian case studies conducted 26 years apart to provide a historical perspective on allied health governance. Although not a longitudinal cohort study, this comparative design tests whether governance challenges recur under similar structural transitions, offering evidence about the persistence or evolution of governance mechanisms that shape workforce experience. The contribution of this research is threefold: (1) it narrows the analytical focus to governance architecture and workforce experience rather than organizational “performance”; (2) it situates Australian findings within international governance debates; and (3) it uses a theoretical frame to explain why matrix tensions endure or dissipate across institutional eras. Accordingly, the following research questions were posed to examine governance tensions across eras:

### 1.1 Research questions

1. How do clinical matrix governance structures shape AHPs workplace experiences in public hospital bed-based services?
2. How and why do specific governance tensions (e.g. accountability, budgetary control, and professional oversight) persist or change across two organizational eras separated by 26 years?

### 1.2 Theoretical framing

This paper delves into the complexities of organizational theory and design, which focuses on understanding how to organize people and resources to achieve the organizations objectives (Greenwood and Miller, 2009; Myers, 1996). Organizational design is dynamic, adapting to contingencies that arise, influenced by structural, human and coordination components (Burton and Obel, 2004). In healthcare, innovative matrix designs like those at John Hopkins and Guys Hospital have led to decentralized clinical units, placing doctors in operational management roles (Heyssel *et al*., 1984). Contingency theory helps explain why hospitals adopt matrix arrangements in resource-intensive contexts (Burton and Obel, 2004). Matrix designs are chosen to reconcile competing demands and improve flexibility and cross-disciplinary coordination (Burton and Obel, 2004; Greenwood and Miller, 2009). However, they introduce coordination costs and ambiguous authority when roles and budgetary control are not clearly aligned (Galbraith, 1971). The shift to matrix structures within hospital settings was notable in the 1990s with AHPs often overlooked regarding their governance needs (Boyce, 1993). Key challenges found by AHPs working in clinical matrix structures included balancing accountability and control while maintaining professional standards and workforce sustainability (Mickan *et al*., 2018; Robinson and Compton, 1996; Turato *et al*., 2023).

Institutional theory foregrounds how enduring rules, norms and professional logics shape organizational structures and resist change (Powell and DiMaggio, 1991). Professional bureaucracy and the sociology of professions literature (Abbott, 1988; Mintzberg, 1979) highlight how jurisdictional boundaries, identity and autonomy affect how professions respond to managerial reforms. Applied to allied health, these lenses explain why matrix reforms that privilege clinical unit control can create professional isolation, role ambiguity and tensions over resource allocation.

Matrix organization theory clarifies the mechanics of dual reporting and the resulting problems of accountability and budgetary alignment (Galbraith, 1971). Relational coordination theory (Gittell, 2009) offers a complementary focus on communication quality and shared goals, which helps interpret findings about perceived inefficiencies and coordination breakdowns reported by AHPs. Combining these lenses enables analysis of both structural arrangements (i.e. control of budgets, accountability) and the relational processes (i.e. communication, trust, shared knowledge) that mediate workplace experience.

This comparative theoretical lens underscores the value of examining how different health systems have approached governance reform, particularly in relation to allied health integration. The following examples briefly illustrate how structural choices, whether integrated, decentralized or matrixed have shaped professional oversight, budgetary control and coordination across varied international contexts.

1. United Kingdom: Integrated Care Systems (ICSs) aim to align providers across pathways but frequently leave profession-specific governance underdeveloped, producing mixed outcomes for allied health integration and voice (Care Quality Commission, 2025; NHS England, 2022).
2. Canada: Regional health authorities decentralize decision-making to enhance local responsiveness; however, decentralization can erode profession-specific oversight unless explicit governance mechanisms are created (Canadian Medical Association, 2025).
3. United States: Large academic medical centers commonly use matrix structures where clinical, academic and administrative lines overlap; studies report recurrent ambiguity for non-physician professional groups when budgetary and operational accountability are misaligned (Wartman, 2024).
4. Scandinavia and the Netherlands: Some reforms have emphasized clinical pathway integration and stronger professional involvement in governance, providing contrasting examples where clearer professional roles were maintained while pursuing integration (European Observatory on Health Systems and Policies, 2024; Maarse and Jeurissen, 2024).

These international variants demonstrate that matrix and decentralized models are not uniquely Australian problems; they are global design choices with similar trade-offs that manifest in allied health governance.

Empirical literature documents workforce shortages, dissatisfaction and recruitment challenges among AHPs, and professional associations have long expressed concerns about governance reforms that dilute profession specific representation (Callan *et al*., 2007; Couch *et al*., 2021; Hewko *et al*., 2019). However, evidence that links specific organizational architectures (especially matrix arrangements) to allied health workforce experiences over extended timeframes is limited. This research addresses this gap in literature by focusing specifically on governance architecture (accountability, budgetary control, professional oversight), professional autonomy and identity, and workforce experience (morale, job satisfaction, role clarity, perceived coordination), this study narrows the conceptual scope and situates its comparative findings within both organizational design and professional governance literatures.

The historically spaced comparative design compares two independent case studies of hospitals transitioning to tertiary facilities 26 years apart. The methodological rationale for this comparative approach is to assess whether governance tensions identified in one institutional moment reappear under similar structural conditions decades later. The analytic aim is to distinguish contingent, context-specific effects from persistent structural or professional dynamics that shape AHPs workplace experiences.

This approach aligns with established traditions of comparative historical analysis, which emphasize the value of temporally spaced cases for distinguishing enduring structural dynamics from context-specific adaptations (George and Bennett, 2005; Pettigrew, 1990; Ragin, 1994). By situating allied health governance within this methodological lineage, the study demonstrates how historical comparison can reveal durable tensions in accountability and professional identity while also tracing how relational processes evolve under shifting institutional conditions.

Changes in broader health system policy, funding environments, workforce expectations and professional norms may influence allied health experiences independently of organizational design. Temporal distance can complicate direct comparison, as shifts in documentation practices, interview conventions and institutional language may affect data richness and comparability. Additionally, the absence of continuous data between the two time points limits the ability to track incremental adaptations or reforms. These limitations are mitigated by the study's focus on structural governance mechanisms rather than performance metrics, and by its use of thematic convergence to identify enduring design tensions across distinct institutional contexts.

Grounded in organizational design and institutional theory, and informed by international examples, this paper examines how clinical matrix governance influences allied health workforce experiences and what governance mechanisms, if any support sustainable, profession-sensitive integration in bed-based public hospital settings. The study intentionally refrains from strong claims about measurable performance outcomes and instead concentrates on workforce relevant governance mechanisms and their implications for policy and organizational design.

### 1.3 Background of each case study

The case studies shared strikingly similar contexts: both involved transitions to newly commissioned tertiary hospital settings and the implementation of clinical matrix structures, which are frequently recommended during hospital restructuring (Fiorio *et al*., 2018). The objectives for adopting the matrix model included enhancing interprofessional collaboration, reducing service gaps, improving operational and strategic planning, demonstrating allied health contributions and providing robust leadership. Despite these aspirations, AHPs in both cases resisted being incorporated into the matrix structure, citing foreseeable operational challenges. Nevertheless, administrators progressed with the model, asserting that potential issues could be managed.

*Case study 1:* Conducted in 1995, this study focused on a multi-site campus hospital in regional New South Wales, Australia, serving a population of 501,650 and housing 642 beds. The hospital was poised to become the teaching facility for a university medical school. The matrix structure was implemented in 1992 and sought to address deficiencies in traditional management by decentralizing operational control to six Patient Care Departments (PCDs), managed by physician unit directors. The matrix structure is illustrated in Figure 1 and shows the six PCDs of medicine, surgery, obstetrics and gynecology, pediatrics, anesthesia and intensive care, and primary/emergency care. Further to the PCDs was the division of corporate and clinical services in which the department heads for each of the main allied health profession disciplines reported to the General Manager of this group. They retained professional responsibility for each profession (e.g. physiotherapy, occupational therapy, social work, psychology, dietetics, speech pathology) but had no operational responsibility. The allied health clinicians working on the front-line were dispersed into each of the six PCDs under medical clinician operational management (Boyce, 1996).

**Figure 1:**
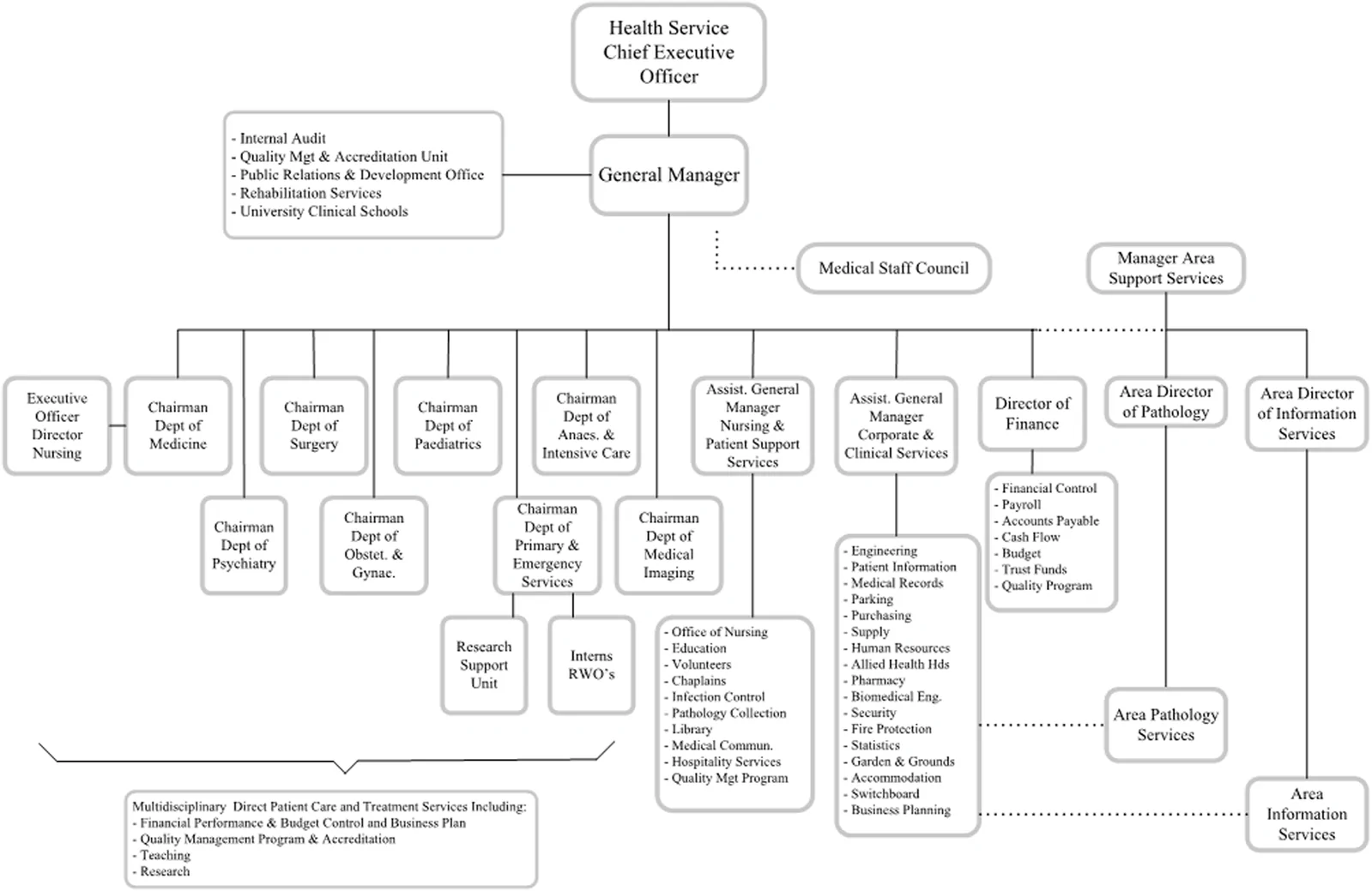
Case Study 1: Organizational chart following alignment to clinical matrix structure

The structure's governance also featured a rotating allied health representative within the executive management committee, contrasting with other committee positions that had permanent tenure. The structure resulted in dual reporting lines for operational (PCDs) and professional (AHPs department heads and allied health representative) accountability for AHPs.

*Case study 2* was conducted in 2018 within a multi-campus hospital and health service in Queensland, serving a catchment of 340,000 people with approximately 700 beds. The hospital partnered with a Queensland University Medical School, welcoming its first medical students in 2019. In preparation for commissioning a new tertiary facility in 2017, AHPs were integrated into a clinical matrix structure starting in 2014.

The re-alignment resulted in the abolition of the allied health executive lead and professional director roles. These were replaced by operational manager roles for Medical and Surgical service groups alongside medical and nursing director roles. A clinical director of allied health role was retained for professional leadership, with professional lead positions established for six therapy professions to provide governance. In 2017, the clinical director role transitioned to an executive of allied health role, which remained a professional position without operational or budgetary responsibilities.

As illustrated in Figure 2, the AHPs aligned to one of five clinical service groups and reported operationally to an allied health manager or lead. The horizontal gray-shaded lines in Figure 2 illustrate the professional lead positions. A consequence of the unit dispersement structure was that AHPs had dual reporting responsibilities. This involved an operational allied health manager for each of the five service groups (i.e. Medical, Surgical, Women's and Children's, Mental Health, Community) to manage the day-to-day operations of allied health staff and an overall professional lead to provide oversight of professional standards and governance of each profession across the entire organization. In both case studies the director of medical imaging and pharmacy retained operational and professional reporting of their workforce.

**Figure 2:**
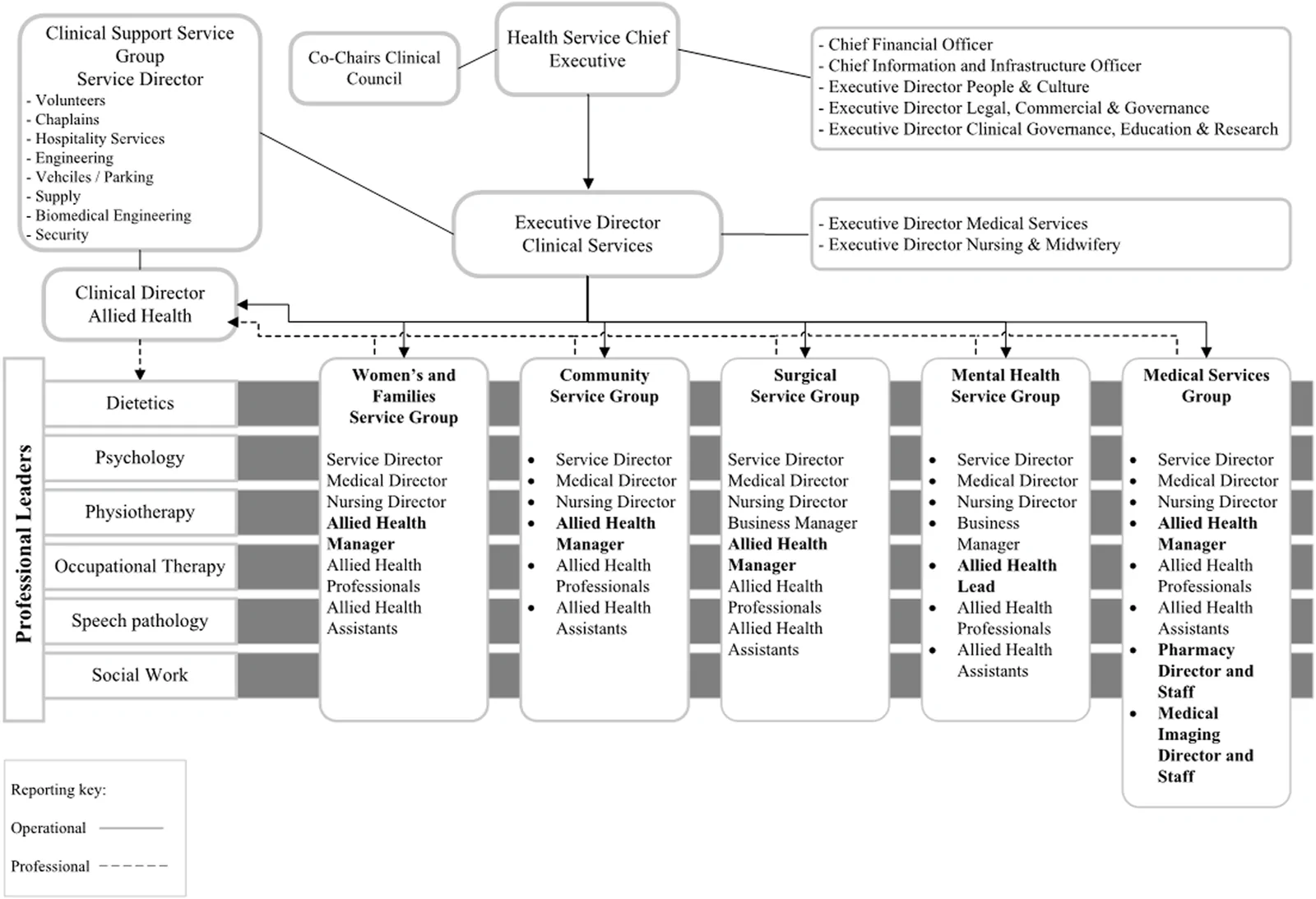
Allied health organizational chart following alignment to the clinical matrix structure

## 2. Methodology

The study provided a unique opportunity to compare raw data files from two independent case studies conducted 26 years apart. The researchers, who had no prior contact, collaborated to conduct a longitudinal comparative analysis, exploring qualitative coding and themes to identify similarities and differences.

### 2.1 Research design

An exploratory research design was employed to observe and gain insights into organizational dynamics following structural re-alignment (Eisenhardt, 1989; Pettigrew, 1990; Zikmund *et al*., 2012). The design was explicitly comparative rather than synthesized, enabling analysis of continuity and change across two independent case studies conducted 26 years apart (Pettigrew, 1990). This approach aligns with established comparative case study methodology, which emphasizes using cases across time to distinguish enduring structural features from contextual variation (George and Bennett, 2005; Pettigrew, 1990). Each case study adopted a qualitative design, featuring in-depth interviews and focus groups to understand participants' experiences with structural changes (Creswell, 2013).

### 2.2 Case study site selection

Literature suggests decision rules for site selection, such as extreme situations, critical incidents and high-visibility cases, which allow the phenomenon under study to be “transparently observable” with strong explanatory utility (Pettigrew, 1990, p. 276). Both case studies qualified as extreme and high-visibility cases due to their comprehensive and prolonged engagement with clinical matrix governance structures (Eisenhardt, 1989). Furthermore, the matrix model is widely recognized as a complex and contested organizational form, particularly in healthcare, where it introduces dual lines of accountability, blurred authority and coordination challenges (Galbraith, 1971; Greenwood and Miller, 2009). These features amplify underlying governance tensions such as conflicts between professional autonomy and managerial control making them more visible and analytically tractable. By selecting cases where the matrix model was deeply embedded, the study was able to explore the structural and relational dynamics of allied health governance with greater clarity, enabling a focus on the deep architecture of organizational experience rather than surface-level variation. The comparative information collected between each of these extreme and high-visibility case studies is summarized in Table 1.

**Table 1:** Comparative data for methods and participants in case studies 1 and 2

Case study 1	Case study 2
Triangulation and multiple methods of data collection	Triangulation and multiple methods of data collection
Interviews: semi-structured – 1 h	Interviews: semi-structured – 1 h Focus groups: 1.5 h Participants: 7 – Executive Managers 4 – Nursing Managers 2 – Medical Clinical Managers 4 – Non-clinical Hospital Managers 30 – Allied Health Manager/Clinical Leader/Professional Lead participated in either an interview (20) and/or focus group (10 each) Total = 47
Participants:	
6 – Executive/General Managers	
2 – Nursing Managers	
2 – Medical Clinical Managers	
6 – Allied Health Profession Managers	
5 – Allied Health Sub-unit Head	
Total = 21	
Audio-taped and transcribed	Audio-taped and transcribed
1200 pages of text	1,115 rows of coded data on excel spreadsheet
Coding process – content analysis	Coding process – content analysis
Themes of inquiry - 17	Themes – 12
Interviews completed by researcher	Interviews completed by research assistant
	Focus groups completed by researcher and co-researcher
Comparative case analysis (against allied health structures in other jurisdictions)	One case study analysed (at a multi-site facility)
Annual report 1993–94	Annual report: 2019–20
New Hospital Commissioned in January 1991	New Hospital Commissioned in March 2017
Registered beds – 642	Registered beds – 700
No senior allied health leadership role. A rotational rep invited onto peak executive meetings	Professional Clinical Director Allied Health role. Not part of executive structure or committees, attended senior leadership meetings
Manager of each allied health profession – No	Manager of each allied health profession – No
Professional lead roles – Yes	Professional lead roles – Yes
Responsible for budget – No	Responsible for budget – No
Allied health manager roles – No	Allied health manager roles – Yes
Direct employment of AHPs by sub-unit – Yes	Direct employment of AHPs by sub-unit – Yes
Background of line manager – Medical	Background of line manager – Medical or Nursing
Professions who participated in research study:	Professions who participated in research study:
Nutrition and Dietetics, Occupational Therapy, Physiotherapy, Psychology, Social Work, Speech Pathology, Pharmacy, Biomedical Engineering	Nutrition and Dietetics, Occupational Therapy, Physiotherapy, Social Work, Psychology, Speech Pathology, Podiatry, Audiology, Orthoptics, Pharmacy, Radiography, Clinical Measurements

### 2.3 Data collection

#### 2.3.1 Case study 1

Recruitment for the study involved asking allied health front-line managers and senior/executive leaders employed at the hospital to voluntarily participate in this research project. The ethical clearance process was conducted over a six-month period and involved obtaining two levels of ethical clearance, one from the hospital in which the study was conducted and the other from the Area Health Board that the hospital reported to. This process was different to the ethical clearance required through a human research ethics committee process in case study 2; however, both were considered robust processes at the time the research was conducted, with the findings previously published in peer-reviewed journals.

A semi-structured approach was incorporated to permit flexibility in the data collection methods for the researcher. The goal of the research was to attain a deep understanding of the case, therefore flexibility in collection methods represents “controlled opportunism” provided it is not conducted in an unsystematic and unpredictable manner (Eisenhardt, 1989). Multiple data collection procedures are an important methodological strategy for demonstrating validity through the use of logical inference and the exercise of judgment grounded in the data to refine constructs and relationships between concepts (Eisenhardt, 1989).

The 17 thematic categories summarized in Table 2, formed the basis for developing a semi-structured interview guide. The interview guide contained a range of items for which data were sought and a set of “probe” questions to stimulate discussion relating to each theme. Twenty-one semi-structured interviews were conducted and audiotaped across executive, nursing, medical and allied health leadership and/or or sub-unit head roles.

**Table 2:** Comparison of the themes of inquiry between case study 1 and case study 2

Theme of inquiry	Case study 1	Case study 2
Vertical reporting	Moderate/Low	Moderate/Low
Managerial independence	Moderate	Moderate/Low
Human Resource flexibility	Low	Low
Lateral communication	Low	Low
Lateral coordination	Low	Low
Fragmentation	High	High
Budget control	Low	Low
Profession-valued factors	Low	Low
Aspirations of professions	Low	Low
Representation	Moderate	Moderate/Low
Collective identity	Low	Low
Integration	Low	Low
Organizational power and influence	Low	Low
Corporate management	Low	Low
Decision-making	Low	Low
Policy and planning	Low	Low
Relationship with medicine/nursing	High	High/Moderate

#### 2.3.2 Case study 2

Recruitment for the study involved an expression of interest request for allied health managers and/or clinical leaders, including senior and executive managers and/or clinical leaders outside of allied health to voluntarily participate in a research project. The study was conducted according to the guidelines of the Declaration of Helsinki and approved by the Prince Charles Hospital, Queensland Health Human Research Ethics Committee in July 2018 (Turato, 2022). An amendment was approved in August 2021 to conduct surveys following a re-structure back to a centralized model. Ethics approval to utilize the data has been maintained through an annual ethics review process conducted in April each year.

For 20 allied health leaders/managers who consented, a 1–1 interview was conducted by a research assistant (i.e. nursing background) to ensure the participants felt comfortable to speak openly and honestly. The interview questions addressed the barriers, enablers and drivers in performing their role within the organization. This included the strategies they believed were necessary for them to perform effectively. The interviews lasted approximately one hour and were audio recorded. Convergence of the data was considered to have been reached after 16 interviews due to no new themes emerging, with four further interviews conducted for confirmation.

After the interviews were transcribed and analyzed, the same 20 participants and an additional ten allied health managers and leaders participated in three (*n* = 10 in each) confirmatory focus groups. A focus group was also conducted with non-allied health executive leaders (*n* = 7) and another with non-allied health senior leaders (*n* = 10), with all focus groups conducted approximately four months after the interviews. The participants in each focus group were presented with the themes from the 1–1 interviews with the allied health managers. Each theme was discussed in detail to confirm if the participants agreed, disagreed or had other comments about the results. Each focus group was managed by the principal researcher and another trained facilitator and lasted approximately two hours. As outlined earlier, the context under study had undergone immense change by transitioning to tertiary level services, which included the commissioning of a new tertiary hospital building. Three years prior to this adjustment was the complete restructure of allied health from a model of professional directors, e.g. Director Physiotherapy, Director Social Work, etc., to a unit dispersement model (Boyce, 2001) under the leadership of allied health managers. The impact of this change was significant, with ongoing escalations of issues and concerns from allied health staff about whether the new structure was effective both operationally and professionally.

Each of the case studies had different theoretical frameworks guiding the questions for the interviews, with case study 1 grounded in sociology and specifically the sociology of professions, with a particular focus on the professional dominance perspective. Specifically, interprofessional relations, organizational arenas and the challenge to the inevitability of AHPs subordination to medicine within the Freidson's construction of professional dominance (Freidson, 1994). Case study 2 utilized the theoretical framework of general systems theory which considers the external conditions that can be beyond one's control which can greatly impact a manager's ability to enact or make a change (Freebairn *et al*., 2017).

### 2.4 Data analysis

Our analytic stance was explicitly comparative across the two independent case studies, using the deductive themes from case study 1 as an anchor to assess continuity and change in case study 2, rather than synthesizing findings into a single consolidated case. Such comparative designs are well recognized in organizational and public health research for their ability to reveal continuity and change across eras (George and Bennett, 2005; Ragin, 1994). To manage the difference in analytic approaches between the two case studies, we ensured methodological transparency and coherence by aligning the coding outputs through shared thematic categories and cross-case comparison. Case study 1 employed structured content analysis, which provided a deductive framework for categorizing data. Case study 2 used reflexive thematic analysis, allowing for greater interpretive depth and responsiveness to emergent meanings. Comparing two different analysis types was challenging due to content analysis being a surface-level pattern recognition approach when compared to reflexive thematic analysis which looks for latent meanings and contextual nuances. To mitigate the challenges of this, we adopted a collaborative reflexivity process to analyze the data. This involved regular team discussions to explore assumptions, challenge interpretations and ensure consistency in theme development. We also maintained detailed analytic memos and used consensus-building techniques to refine and validate the thematic structure, balancing individual insights with collective rigor (Humble and Mozelius, 2022). The analysis methodology is outlined for each case study next.

#### 2.4.1 Case study 1

The text from all recorded interviews was transcribed by electronic means to enable later manipulation by computer. The electronic record was subjected to content analysis and coded according to the 17 thematic categories (i.e. listed in Table 2) and other emergent categories (Schreier, 2012). The content analysis was an iterative process as new coding categories were created, and prior coding re-assessed in light of the codes which were created. A segment of text might be coded against more than one category. The transcripts from the interviews were analyzed using a manual coding process in which a theme of inquiry was extrapolated via a triangulation process from a literature review, international fieldwork investigation of hospital organization and interviews with key profession leaders. Following the identification of indicators from the triangulation of data sources referred to above, and their modification for use in the Australian setting, we applied a three-tier classification (“low”, “moderate”, “high”) to indicate each theme's relative prominence, importance and impact within the case, enabling transparent comparison across eras (importance and impact here refer to interpretive weight rather than statistical significance or frequency counts). The design of the data classification system followed the recommendations offered by Miles and Huberman for delineating the minimum conditions for data reduction strategies in the production of matrices (Miles and Huberman, 1994).

#### 2.4.2 Case study 2

The transcripts from the interviews were thematically analyzed using a manual process involving a six-phase thematic analysis methodology (Braun and Clarke, 2022). The first phase involved migrating the data from the interviews to a Microsoft Excel worksheet, generating a single column consisting of all comments. The second phase included generating initial codes by reviewing each individual cell (participant comment) and assigning a potential thematic area, to which a cell color code was applied (e.g. yellow, blue, orange, grey). Using the Excel sorting tool, the interview data was sorted by the color assigned to each cell and therefore sorted by thematic area. In phase three. the researchers analyzed and interpreted the data by copying the data to a second Excel worksheet and completing a “first pass” over the data, by grouping similar comments to consolidate the data. Every time the pass was performed for each thematic area, the data were moved to a new worksheet. Phase 4 involved a two-researcher confirmation approach, in which each stage of the data analysis was reviewed. Phase five defined and named the themes, which started once both researchers were satisfied with the thematic map of the data. The final phase included reporting the themes and presenting these to the focus group participants for further clarification and verification. The above phases were repeated during the analysis of the focus group data to ensure the overall themes were confirmed and captured accurately, providing a robust basis for cross-case comparison against the deductive framework from case study 1.

The researchers carefully reviewed the data from case study 2 and compared this against the 17 themes of inquiry extrapolated in case study 1 and followed the below six-step process.

1. Theme extraction: Seventeen themes of inquiry were extrapolated from case study 1 using structured content analysis.
2. Data review: Transcripts and coded data from case study 2 were reviewed using reflexive thematic analysis.
3. Theme mapping: Findings from case study 2 were mapped against the pre-established themes from case study 1.
4. Comparative ranking: Each theme was assessed for its prominence and impact in case study 2 using a three-tier scale (low, moderate, high).
5. Consensus validation: Rankings were discussed and refined collaboratively among the research team to ensure consistency and interpretive rigor.
6. Tabular presentation: A summary table (Table 2) presents the final rankings across both case studies to support transparency and cross-case comparison.

## 3. Findings

The findings address the study's focal questions by reporting results from a cross-case analysis of two Australian public hospital organizations that used allied health clinical matrix structures. Using six thematic headings, we present the empirically grounded themes and subthemes identified by two independent researchers working 26 years apart, supported by participant quotations and comparative ratings and factual temporal contrasts in Table 2 that indicate which governance features persisted or changed over time.

### 3.1 Human resource management, reporting, communication, coordination, fragmentation

The clinical matrix structure produced blurred boundaries between operational and professional governance, creating persistent managerial challenges for allied health leaders across both cases. Dual reporting lines and competing clinical priorities were consistently reported and described by participants as generating “structural tension” for managers, with one participant stating, “you have to negotiate … They will say ‘we are going to keep our staff … We pay for them and we want them’” (Case Study 1, allied health sub-unit head).

Staff stress, workload imbalances and workforce fragmentation were frequently observed, with one participate noting, “staff are refusing to help others … some staff are run off their feet and others are putting their feet up” (Case Study 2, allied health manager). Another commented, “no one really is going to pitch in … They may want to, but they are not meant to” (Case Study 1, allied health sub-unit head). Fragmentation encouraged specialization and service ownership that aligned to unit funding rather than broader patient-level priorities, and multiple reporting lines increased bureaucratic complexity and administrative burden. Participants linked these structural features to a need for clearer accountability: “the current dual reporting structure is confusing with several masters … we need single points of accountability” (Case Study 2, allied health manager). Both studies found that dispersing AHPs into clinical units amplified tensions, reduced workforce flexibility and complicated cross-unit coordination.

### 3.2 Control of budget and costs

Control over profession-specific resources was experienced as a persistent problem across both cases. Participants repeatedly described operational inefficiencies and administrative burden arising when allied health professional leaders lacked direct budget authority. For example, one participant observed, “The human resource establishment structure needs to change to ensure allied health can flex its workforce easily without the complexity of forms” (Case Study 2, professional lead), and another noted, “The current structure of allied health increases the clinical leader's administrative workload and therefore they have less time to work clinically or support their staff” (Case Study 2, clinical leader). In Case Study 2 many allied health positions were split across multiple position numbers (sometimes up to four) to allocate costs across clinical units; participants linked this arrangement to increased payroll errors, time-consuming negotiations and higher administrative overhead. One participant expressed, “If we could relieve the pressure of administration tasks and streamline processes, it would allow leadership more time to do the leadership aspect of their roles rather than all management” (Case Study 2, professional lead).

Both studies also documented inequities in professional development funding when clinical units managed these budgets independently, producing frustration and turnover risk, which one comment highlighted, “If the support is not there for them to have any professional development … They leave, and the ones that stay are in the supportive PCDs” (Case Study 1, head of discipline). Administrative complexity sometimes resulted in cancelled clinical rotations and loss of clinical time, with comments like, “Senior allied health staff are losing clinical time to admin time” and “Each staff member should be working as they should be … Admin doing admin and clinicians being clinicians, it's so wasteful” (Case Study 2, professional lead). In both cases, dispersed control over budgets led to persistent inefficiencies, increased administrative demands, and dissatisfaction among staff, while constraining professional governance and workforce responsiveness.

### 3.3 Profession valued factors and aspirations

Both case studies reported inequities in access to professional development, research opportunities and clear career progression for AHPs. Research activity was effectively absent in Case Study 1 and described as difficult to progress in Case Study 2 because clinical units controlled the relevant budgets. Participants framed these constraints as time-consuming and disheartening, with comments like, “You spend an inordinate amount of time on this model lobbying, politicking, counting the numbers … trying to get consensus by all those other processes” (Case Study 1, Nurse Manager). The reduced ability for career progression impacted retention of staff with one participant stating, “Our junior staff are leaving as there are no opportunities to rotate between medical and surgical clinical units, and they don't want to stay” (Professional lead, Case Study 2).

Performance appraisal practices were reported as inconsistent and fragmented in Case Study 2 with appraisals requiring input from multiple managers, creating delays and unmet aspirations, with one comment summing up the participants perceptions, “Performance appraisals are difficult to organize and keep on track, due to needing to book these with several operational managers … who often have no idea about the performance of the AHPs they are managing” (Professional lead, Case Study 2). In contrast, Case Study 1 appraisals were performed solely by professional leaders, leaving some staff feeling unsupported: “I have no idea what the PCD feels about my performance … you do not receive that sort of support (performance appraisal) from the departments” (Case Study 1, head of discipline).

Both studies linked restricted funding and fractured governance to reduced innovation and a diminished sense of professional autonomy; one head of discipline summarized this disillusionment, “You have been conned into thinking that you have autonomy, power, and control. All you have is an impression of influence within a very limited sphere” (Case Study 1, head of discipline). These patterns were associated with reduced job satisfaction and retention risk across both cases.

### 3.4 Allied health representation, collective identity, organizational power and influence

Both case studies reported that commissioning decisions for new tertiary facilities favored clinical matrix designs without systematically considering governance models tailored to allied health, and participants perceived this as a key upstream driver of downstream governance problems. Within the matrix, professional reporting lines were routinely experienced as secondary to operational lines, with comments like, “It's difficult … the operational managers ultimately have the authority to make decisions … the professional line is not seen as important” (Case Study 2, professional lead). Reporting to managers from different professions was commonly described as alienating, which this comment sums up, “Reporting to someone of a different profession … is hard, staff feel unheard” (Case Study 2, professional lead). Participants linked these dynamics to practical governance barriers affecting recruitment, rotations, education and innovation.

Multiple respondents described a weakened collective allied health voice and excessive effort required to secure attention at organizational forums, with comments like “the meetings capital of the world!” (Case Study 1, head of discipline) and “there is a loss of overall voice for allied health” (Case Study 1, various participants). Senior allied health representatives were frequently reported to lack formal authority over budgets or staff, limiting their capacity to defend professional interests: “If you don't have power and financial responsibility … it is more difficult to defend” (Case Study 1, PCD Chairman). Participants described the structure enabling autonomous “mini-hospital” behavior in clinical units, producing insularity and misalignment with corporate objectives, with one participant stating, “a lot more effort is required to make things work because clinical goals often clash with corporate ones” (Case Study 1, executive leader).

Professional identity and collective purpose were reported to suffer, with consequences for morale and retention. One participant commented, “it is siloed and professions have lost their identity by being cut up into small pieces, staff then only look at their small piece” (Case Study 2, professional lead). Both studies concluded that fragmented representation and weak organizational influence constrained allied health's capacity to shape strategic planning, standards of care and cross-unit collaboration.

### 3.5 Corporate management, decision making, policy and planning

Participants in both case studies reported limited allied health representation and influence in corporate management, strategic decision making and policy planning. Allied health leaders described restricted access to top-level committees, weak advocacy for allied health priorities and inadequate resource allocation, with one PCD Chairman observing, “Advocacy for allied health has not been strong enough … allied health is often at the bottom of the priority list” (Case Study 1, PCD Chairman). Representation often depended on indirect validation from medical leaders rather than direct allied health authority, and participants linked this to difficulties securing collective positions on strategic matters.

A Director of Nursing described the managerial fragmentation as multiplying governance problems: “You have so many tribes … dispersed into clinical units with no clear management or professional support. It's ten-fold the problem of what nursing and midwifery has from a tribal perspective” (Case Study 2, Director of Nursing). Strategic planning for core allied health functions such as designing clinical models of care, staff development, financial management and research activity was reported as largely absent, constraining innovation and organizational contribution. Participants called for more open executive allied health dialogue, with comments like, “More open discussion between executive and allied health is required about the structure and the most efficient way to deliver great outcomes” (Case Study 2, professional lead). Some local positive relationships with medical and nursing colleagues were noted, but overall both studies concluded that the matrix arrangement limited allied health's corporate voice and capacity to shape policy and planning.

### 3.6 Relationship with medicine and nursing in the clinical units

Senior AHPs who were embedded as “in-charge” clinicians within clinical units reported high job satisfaction derived from close working relationships with medical and nursing colleagues, direct collaboration, professional recognition and a strong sense of belonging. Participants contrasted this local satisfaction with the challenges faced by Heads of Discipline and Professional Leads who had responsibility for organization-wide profession issues but lacked the resources and authority to effect change: “The In-Charge allied health seniors feel part of the clinical team … they can actually do their jobs; they can also specialize. The difficulty is that the notional heads of each discipline … have to deal with six or seven different allied health senior leaders in the clinical units, all who have different priorities, and that is really difficult” (Case Study 1, head of discipline).

Both case studies documented that the matrix model supported patient-focused care and enhanced interprofessional teamwork at the unit level, but simultaneously produced stronger local professional autonomy and siloing. Participants described behaviors that protected unit interests at the expense of organization-wide coherence, with comments like, “The current allied health structure does not fully optimize the workforce. It's not looking at the organization as a whole; it's a siloed approach … Staff are acting out when a decision does not go their way and causing drama to try and influence change and protect their needs” (Case Study 2, professional lead). Overall, the evidence shows a consistent pattern that local integration with medicine and nursing improved immediate teamwork and job satisfaction for unit-based AHPs, while weakening unified allied health perspectives and complicating the work of Heads of Discipline and/or Professional Leads responsible for system-wide planning and standards.

## 4. Discussion

This study investigated how clinical matrix governance structures shape AHPs workplace experiences in bed-based public hospital services. Using two qualitative case studies conducted 26 years apart, it explored persistent governance tensions (e.g. accountability, budgetary control and professional oversight) across the decades and their implications on the allied health workforce.

Interpreting the cross-case findings through matrix organization theory and relational coordination reveals a clear and consistent pattern: structural choices that disperse budgetary authority and prioritize operational reporting create accountability gaps, increase administrative overhead, weaken professional governance and fray relational processes. Together, these dynamics constrained allied health capability and morale across both hospitals, suggesting a durable design-level misfit rather than transient managerial failures. To strengthen the contribution of these findings, it is important to reflect on how our comparative design across two eras adds methodological value to allied health governance research.

Our decision to adopt a comparative rather than a synthesized approach contributes methodologically by demonstrating how historical case analysis can reveal both durable governance tensions and context adaptations in allied health (George and Bennett, 2005; Pettigrew, 1990; Ragin, 1994). Using a deductive thematic framework from an earlier era as an analytic anchor, and then examining latent meanings in a later era through reflexive thematic analysis (Braun and Clarke, 2022), allowed us to differentiate structural continuity from temporal change. This cross-era design clarifies when managerial logics and professional identities persist despite reform, and when they are reshaped by organizational conditions, offering a replicable template for workforce governance research across time (Burton and Obel, 2004).

In both cases, overlapping authorities emerged when budget holders were embedded in clinical units while professional leaders retained responsibility for standards and workforce stewardship. That split prompted clinical units to protect funded posts and resist workforce reallocation, encouraged position-splitting and multiple cost centers, and drew allied health leaders into disproportionate administration and negotiation. The consequence was higher transaction costs, including increased payroll errors, repeated bargaining, duplicated effort and the diversion of senior clinicians from strategic and clinical leadership to operational tasks (Boyce, 1993; Haas and Hall, 1996; Ovretveit, 1992).

These structural misalignments also undermined relational coordination. Communication became fragmented, shared goals weakened and problem-solving was delayed; mutual trust eroded and manifested in refusals to assist across units, canceled rotations and reduced collaborative innovation (Gittell, 2009). The combined effect of perverse structural incentives and frayed relationships amplified workforce fragmentation and reduced flexibility, thereby constraining surge capacity and responses to patient-flow pressures.

Dispersed budget and decision rights limited allied health leaders' control over professional development, research and career pathways, undermining innovation and retention (Boyce, 1993; Hewko *et al*., 2019; Stagnitti *et al*., 2006). Weak formal representation at corporate tables produced tokenism and a diminished collective allied health voice. Social identity theory helps explain why strong local identification with clinical teams – while beneficial for unit-level collaboration – reduces commitment to organization-wide standards and collective strategic engagement, producing a trade-off between local job satisfaction and diminished capacity for profession-level leadership (Ashforth and Mael, 1989; Burton and Obel, 2004).

Eroding profession-based hierarchies also curtailed direct access to corporate planning, leaving allied health marginalized in models of care, workforce development, financial planning, and research and innovation strategy (Boyce, 2001; Mickan *et al*., 2018; Ovretveit, 1992; Porter and Wilton, 2020). Reliance on medical validation for advocacy and the absence of resourced, authorized allied health strategic roles embedded at the executive level meant allied health priorities were often under-represented or deprioritized at that level.

At the same time, embedding allied health professionals into clinical units produced tangible local benefits such as stronger day-to-day interprofessional collaboration, clearer recognition and improved teamwork. However, without governance mechanisms that translate local innovations into system standards, these gains tended to reinforce local optimization and siloing, producing “mini-hospital” behaviors that complicated discipline-level efforts to maintain coherent standards and equitable workforce policies (Boyce, 2006; Turato *et al*., 2023).

### 4.1 Implications for governance reform

Cross-case convergence across six thematic domains highlights two complementary policy pathways. The first is structural reconfiguration to correct design-level misalignments, and the second relational strengthening to rebuild coordination and trust.

Structural reforms should reduce budget fragmentation (e.g. limit position-splitting) and establish a centralized allied health governance function with delegated authority, enabling professional leaders to make workforce and career development decisions. Formal, resourced representation at the executive level is essential to secure profession-level governance and strategic voice (Boyce, 1993; Haas and Hall, 1996; Ovretveit, 1992).

Relational and procedural reforms must proceed in parallel. Standardized rostering and payroll protocols, joint appraisal calibration, cross-unit coordination forums and shared performance measures can rebuild timely communication, align goals and reduce repeated bargaining (Gittell, 2009). A balanced design preserves the relational benefits of embedding AHPs in clinical units while introducing explicit mechanisms such as clear role descriptions, protected time and mandated participation in discipline-level planning that translate local innovation into organization-wide standards and reduce tokenistic representation (Burton and Obel, 2004; Turato *et al*., 2023).

### 4.2 Practical recommendations for hospital leaders and policymakers

To operationalize these governance reforms, we propose the following actions:

1. Recognize structural design as a key determinant of allied health capability, workforce well-being and governance effectiveness.
2. Require evidence that major redesigns (e.g. matrix models) will safeguard profession-level governance and workforce sustainability before implementation.
3. Centralize allied health budgets and professional authority to reduce fragmentation and administrative burden, while retaining operational embedding to support unit-level collaboration.
4. Appoint an executive allied health lead with delegated budgetary and decision-making rights, supported by embedded roles within clinical pathways to maintain local integration.
5. Treat allied health governance as a distinct design responsibility, with allocated authority, resourced leadership roles and measurable corporate accountability.
6. Fund dedicated workforce development and conjoint research roles to drive training, innovation, cross-unit coordination, performance frameworks and research translation.

### 4.3 Comparative and policy relevance

International and disciplinary evidence shows that decentralization or integration without profession-level safeguards risks repeating the deficits observed here; conversely, systems that combine integration with protected professional governance and resource control provide constructive models for reform (Boyce, 1993; Mickan *et al*., 2018; Turato *et al*., 2023). For policymakers, the principal lesson is that structural change (how authority and budgets are allocated) matters as much as culture change (how people communicate and build shared goals). Effective governance reform therefore requires paired structural and relational interventions so that local clinical advantages do not come at the expense of profession-level leadership, workforce flexibility or system coherence.

### 4.4 Directions for future research

Future research should build on past research findings that have demonstrated positive organogram outcomes from implementing a centralized allied health structure within hospital bed-based services (Turato *et al*., 2024). Future research needs to embed mixed-methods evaluation and implementation science approaches to identify barriers and enablers for allied health governance structures. Comparative and quasi-experimental studies could assess effects on retention, patient outcomes and costs, while micro-level and co-design research should unpack the mechanisms (budget control, appraisal ownership, redeployment authority) needed to deliver scalable and acceptable reform.

### 4.5 Limitations

The study relied exclusively on interviews with leaders and managers, which provides a coherent governance perspective but omits frontline AHPs, support staff and patient voices; this single-stakeholder sampling risks perspective and power bias, retrospective or social-desirability effects, and limits insight into day-to-day clinical practice and patient experience. The absence of methodological triangulation (no frontline interviews, observations or administrative outcome data) constrains causal claims about operational or patient-level impacts and limits transferability beyond bed-based allied health in similar public hospital settings due to the qualitative case approach limiting generalizability. Future work should include frontline and patient perspectives, direct observation and quantitative metrics to validate and extend these leader-centered findings.

## 5. Conclusion

This study was undertaken to critically examine how organizational governance structures; specifically clinical matrix models shape the capacity of AHPs to deliver high-quality patient care within public hospital settings. By comparing two case studies conducted 26 years apart, the research sought to illuminate the enduring and evolving implications of matrix governance on allied health performance, morale and financial stewardship, thereby contributing longitudinal insight to a field where such evidence remains scarce.

This cross-case comparison shows that the clinical matrix governance structures implemented in the two Australian hospital systems systematically produced enduring tensions for AHPs such as fragmented accountability, misaligned budgetary authority, weakened professional voice and strained relational coordination. These structural features consistently undermined workforce flexibility, innovation and morale across time.

To prevent recurrence while preserving the local benefits of embedding AHPs (strong interprofessional relationships and patient-centered collaboration), hospitals should pair unit-level integration with explicit governance safeguards that restore clear decision rights and formal allied health authority, and that embed cross-unit communication and shared goals needed for integrated, equitable and sustainable service delivery.
